# Analysis of the medical residency matching algorithm to validate and improve equity

**DOI:** 10.1371/journal.pone.0284153

**Published:** 2023-04-06

**Authors:** Briance Mascarenhas, Kartikeye Puranam

**Affiliations:** Management Area, School of Business, Rutgers University, Camden, New Jersey, United States of America; University of Malta, MALTA

## Abstract

Algorithms are becoming prevalent but are often opaque and need external validation to assess whether or not they meet their purported objectives. The purpose of this study is to validate, using the limited information available, the algorithm used by the National Resident Matching Program (NRMP) whose intention is to match applicants to medical residencies based on applicants’ prioritized preferences. The methodology involved first using randomized computer-generated data to overcome the inaccessible proprietary data on applicant and program rankings. Simulations using these data were run through the compiled algorithm’s procedures to obtain match outcomes. The study’s findings are that the current algorithm’s matches are related to program input but not to applicant input, the applicant’s prioritized ranking of programs. A modified algorithm with student input as the primary factor is then developed and run using the same data, resulting in match outcomes that are related to both applicant and program inputs, improving equity.

## Introduction

With the rise and spread of computing power, algorithms have become prevalent and process large volumes of transactions in diverse functions and organizations. The algorithms intend to institute standard operating procedures, reduce transaction costs, and provide benefits for the purveyors, adopters, and users of these algorithms.

In healthcare delivery, algorithm protocols compared to physician decision-making may play a beneficial role by alleviating racial biases by treating everyone equally through standardized methods and have been found to reduce the incidence of amputations in favor of revascularizations for black patients [1].

Over time, however, concerns have been raised about unconscious algorithm bias that may perpetuate and exacerbate social inequities. Algorithms are designed often to serve mainly the needs of the privileged, and their performance or procedures cannot be adequately scrutinized [2,3]. Algorithms are often black-boxes that face challenges in validation, regulation, and integration [4]. They are often opaque, not based on appropriate or representative data, too complex to understand, may not achieve what they promise, and are not tracked for improvement over time [4].

A widely used algorithm was found to be biased against Black patients because it used health costs as a proxy for health needs, and Black patients spend less money than other patients for the same level of need [5]. Similarly, an algorithm that simply optimizes cost effectiveness in ad delivery was found to deliver gender-neutral ads in an apparently discriminatory way to young women, because of crowding out by other ads to this prized targeted group [6].

Algorithmic bias is widespread across a variety of contexts, and will increase with the growing prevalence of algorithm use for analysis of big data, creating an increasing need for their validation There have been calls for algorithms to be validated with respect to whether or not they meet an organizations clinical needs [7], whether or not they deliver on their intended purpose; the extent to which they match the organization’s education, skills, and regulatory competencies [8,9]; data inputs [10], cost budget constraints [11]; their prediction accuracy [12,13], performance when applied to diverse subgroups of the population [13,14], having continuous monitoring and recalibration [14], fairness trust and transparency [15], and whether or not they meet the larger goals of fairness, ethics, social equity, inclusion, access, and diversity [16]. The United Nations Sustainable Development Goal 10 seeks to reduce societal inequalities, within and between nations [17].

Algorithms, however, are often not validated by practitioners. “How to ensure that the algorithm is fair, how to make sure the algorithm is really interpretable and explainable—that’s still quite far off” [18]. Fairness is relevant for legal compliance, social responsibility, and utility: unfair algorithms create social harms and threaten an organization’s survival, competitiveness, and overall performance [19].

Four possible areas to address bias in practice are: 1. At the problem formulation stage, 2. At the data level, 3. Through modification of the algorithm, and 4. By post-processing the output [20]. Stakeholders have limited access to the algorithm’s data and processes, impeding its analysis. Transparency would be helpful to examine and uncover possible biases in algorithms, but vendors are often not particularly forthcoming about their practices [21]. This limited transparency and access pose fundamental challenges to identifying and mitigating an algorithm’s possible bias. Bias needs to be reduced to improve an algorithm’s accuracy and inclusion. Modification of an imperfect algorithm by giving users even a small amount of control has been found to dramatically increase users’ willingness to use it, confidence in it, and benefits from it [22].

This article examines the algorithm used by the NRMP that matches more than 60,000 medical graduates to more than 30,000 residency positions in over 4000 hospital programs annually. The matching algorithm that is currently used was last revised more than a generation ago [23,24] and the majority of recent applicants (60%) feel that the process needs to be changed [25].

Each medical graduate submits to the NRMP her/his ranking of programs to which they have applied. Similarly, each program submits to the NRMP its ranking of applicants. The NRMP’s algorithm has been called applicant-proposed because it first tries to place an applicant into her/his most preferred program, subject to the program’s number of residency positions available. Initial matches are tentative, and a student may be dematched in a subsequent iteration if that program ranks another student higher. The matching algorithm runs until all applicants and programs are considered. The NRMP intends to use an algorithm that prioritizes the preferences of applicants, placing them in the most preferred program possible on their rank ordered list [26].

The main objective of the matching algorithm is to fill residency positions by matching applicants to programs based on applicants’ prioritized preferences. Students have long raised concerns, however, that the current algorithm favors programs over students [24,27]. Students are small players with few resources and experience relative to programs, and so may be at a disadvantage when using the algorithm. Compared to the current program-centric algorithm, a student-centric algorithm was able to match more students into their first-choice and top-five-choice programs [28].

There is a research gap in accessing and validating algorithms in health care. Machine learning for health compares unfavorably to other areas regarding reproducibility metrics such as dataset and code accessibility [29]. The inputs to the current residency matching algorithm, the individual program and student rankings, are kept private. How the NRMP algorithm works is also confusing to many students and program directors [25,30]. The algorithm has continued to be used for decades without modification or innovation. There is a perception that the current algorithm’s matches are stable and optimal. A perception of algorithm complexity does not discourage its use [31]. When information access is limited, the verification of possible algorithm bias and its modification to reduce the bias poses major difficulties and challenges, resulting in a research gap.

This study of the medical residency matching algorithm is novel in that it shows how the widespread problems of inaccessibility to input data and limited information about the algorithm’s procedures can be overcome. It introduces a statistical method for measuring the relative benefits that various stakeholders derive from the algorithm. Finally, it shows how an algorithm can be modified to move towards being more beneficial to a disadvantaged stakeholder.

## Materials and methods

**Fig 1:**
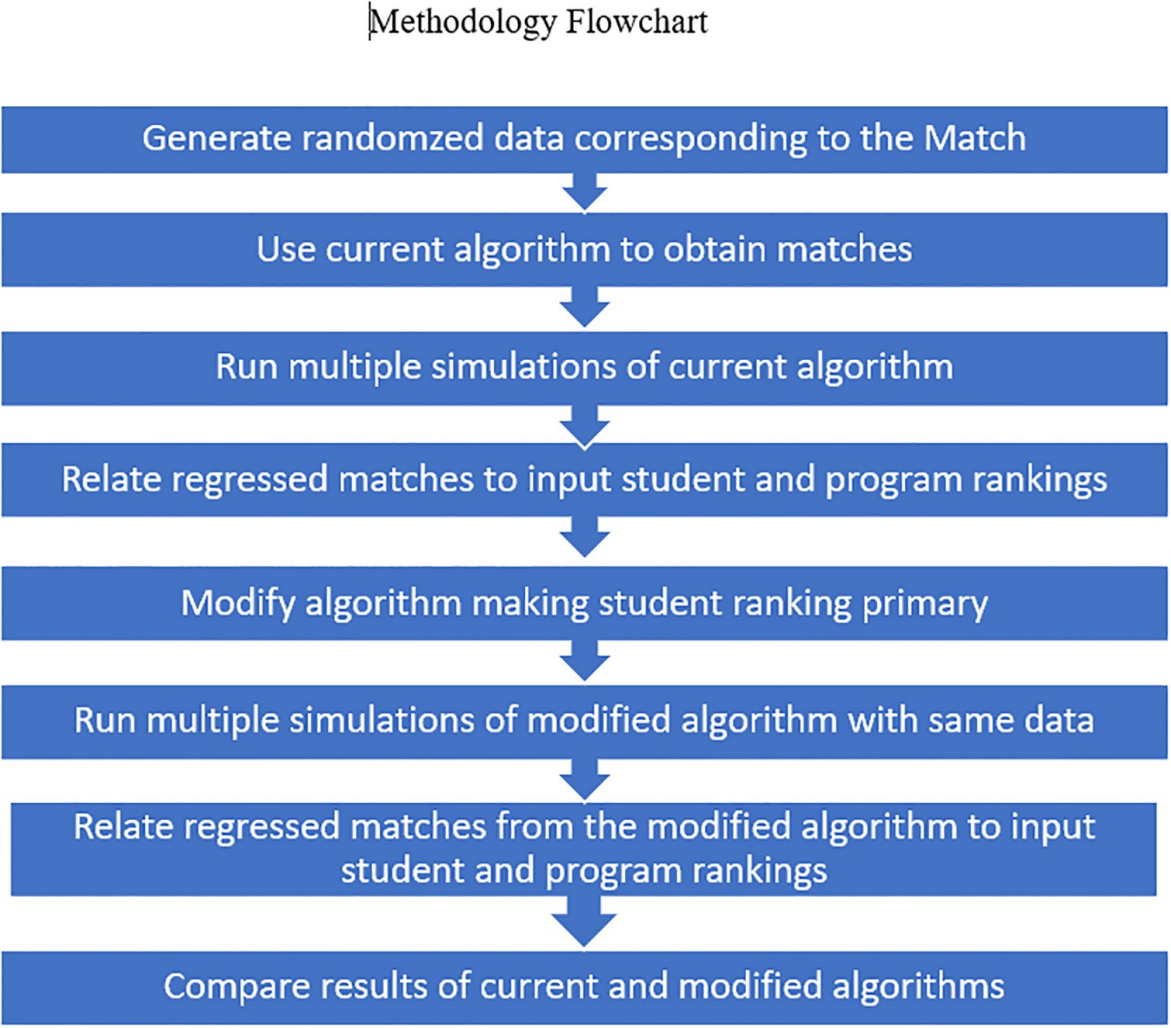
The flowchart below summarizes the methodology.

The NRMP provides limited information about the annual Match. It provides aggregate statistics on the annual residency match and a video description of how the algorithm seeks to match five students and three programs [32,33]. It does not reveal the individual student and program rankings and their match outcomes, which are private Fig 1.

Using computer-generated randomized data helps to cover a broad range of variable values. Student rank order lists ranged between 0 and 20 programs, ranked in random order. Program rank order lists were generated with lengths between 60 and 90 students, ranked in random order. The program lists are generated to be consistent with the student lists, i.e., only those students that ranked by a particular program are in turn ranked by the program. We ran 2600 simulations of the algorithm using the randomized student and program rankings data to determine match outcomes. All simulations were done in Python version 3.9.12. The researchers ran the current NRMP algorithm using the software code available in Henry Wilde’s matching library [34] to obtain match outcomes. A match outcome is defined as whether or not a specific student matched to a particular program.

Next, the study performed multiple logistic regression of the binary dependent variable, whether or not a student matched to a program, against the focal independent student and program ranking variables in order to examine their influence. If the algorithm is fair, both the student and program rankings are expected to be significantly related to the probability of a match. The logistic regression included additional independent control variables–length of a student’s rank ordered list, length of the program’s rank ordered list, number of positions available in a program, and interaction variables. Related to a bivariate analysis, a logistic regression analysis with multiple linear and non-linear independent variables provides a more stringent test for the existence or absence of a relationship between the data inputs and dependent variable.

After finding that the match outcomes were related to program rankings, but not student rankings, the researchers modified the current algorithm. In the current algorithm, students’ rankings are considered first but matches are tentative. Program rankings are then used to determine if a match is permanent and to break ties among students. Thus, program rankings are the primary factor in the matching decision and break ties among students. The algorithm was modified so that student rankings are the primary factor in attempting a match, while the program rankings are the secondary factor and used to break ties among students. The revised algorithm was developed by the researchers in Python. Logistic regression analyses were conducted using R version 4.2.2.

Using the same data used in the current algorithm, 2600 simulations were also run with the modified algorithm to determine match outcomes. Running the same data across the two algorithms, controls for the data, and focuses the analysis on the different procedures across the two algorithms. The match outcomes were again logistically regressed against the student and program rankings, as well as the control independent variables. The coefficient estimates for the program-centric and student-centric algorithm models were examined to assess their relation to the match outcomes. We examined the coefficients and the odds-ratios for the program ranking and student ranking variables, comparing them within an algorithm model and across the two algorithm models.

## Results and discussion

Table 1 reports the numbers of programs, the number of residency positions, and the number of graduate medical student applicants in the 2021 match program.

**Table 1:** National resident matching program number of programs, applicants, and positions in 2021.

	Total
Number of Programs	5,136
Number of Positions	35,194
Number of Applicants	63,990

Table 2 shows the multiple logistic regression results with match outcome as the binary dependent variable. The table shows the variable coefficients and below them the standard errors in brackets. The second row shows that with the current program-centric algorithm model the program’s ranking of students (-0.447) is significant at the 0.01 level, with a lower (better) rank being associated with a higher probability of a match. The student’s ranking of a program (0.009), however, is not significant even at the 0.05 level and shows no relation with the probability of a match. Thus, the regression model results provide statistical evidence that the current algorithm favors program input over that of students, and provides no evidence that the algorithm prioritizes the preferences of applicants and places them in the most preferred program possible on their rank ordered list.

**Table 2:** Multiple logistical regression models of matching.

Model:	Student Rank Ordered List Length	Student’s Rank of Program	Program’s Rank of Student	Program’s Number of Positions	Interaction Student List x Number of positions	Interaction Student’s rank x Program’s rank	n	Hosmer & Lemeshaw R-Sq
Current	0.041** (0.006)	0.009 (0.011)	-0.447** (0.004)	0.746** (0.01)	0.007** (0.01)	-0.003** (0.001)	205,763	0.80
Modified	0.08** (0.006)	-4.98** (0.047)	-0.135** (0.002)	0.186** (0.006)	0.007** (0.001)	0.05** (0.001)	161,589	0.63
Coefficient difference	-0.038** (0.009)	4.991** (0.048)	-0.312** (0.005)	0.56** (0.011)	0.0** (0.001)	-0.053** (0.001)		

*Significant at 0.05 level.

**Significant at the 0.01 level.

The third row shows that in the modified algorithm model the program’s ranking of students (-0.135) remains significant at the 0.01 level, but now the student rankings coefficient (-4.991) become significant at the 0.01 level, suggesting that a lower (better) program ranking and student ranking are associated with a higher probability of a match. Thus, modifying the roles of student and program rankings in the algorithm has reduced the current algorithm’s bias and has enabled both programs and students to have a significant influence on the matching outcome.

Table 3 shows the odds ratios for the two algorithm models. An odds ratio close to 1 suggests little change in the probability of a match with a change in that variable. A ratio substantially above 1 and below 1 suggests substantial increases and decreases in the odds of a match, respectively.

**Table 3:** Algorithm models—Odds ratios of matching.

Model:	Student Rank Ordered List Length	Student’s Rank of Program	Program’s Rank of Student	Program’s Number of Positions	Interaction Student List x Number of positions	Interaction Student’s rank x Program’s rank	n	Hosmer & Lemeshaw R-Sq
Current	1.04**	1.01	0.64**	2.10**	1.01**	1.00**	205,763	0.80
Modified	1.08**	0.01**	0.87**	1.20**	1.01**	1.05**	161,589	0.63

*Significant at 0.05 level.

**Significant at the 0.01 level.

In the second row, the current algorithm’s model shows that a lower (better) program’s ranking (0.64) significantly (at the 0.01 level) improves the odds of a match. A change in student’s ranking of the program (1.01) has no effect on the odds of matching, however. Thus, the main purpose of the matching algorithm to take into account both the preferences of students and programs is not being achieved.

In the third row, the modified algorithm’s model shows that both the program’s ranking of students (0.87) and student’s ranking of programs (0.01) odds ratios are below 1 and significant at the 0.01 level. Thus, the bias in the current model has been reduced in the modified model, where both students and program rankings influence the odds of a match.

The table results also show that program’s number of positions increases the odds of a match in the current model (2.10) and in the modified model (1.20). The odds of a match can be increased by students applying to programs offering more positions, and by programs offering more positions. The rest of the variables have odds ratios close to 1, suggesting little relation to the probability of matching.

Finally, row 4 in Table 2 shows that the two algorithm models are significantly different from each other at the 0.01 level with respect to each of the coefficients. However, the overall match performance, the percentage of all applicants matched to positions, of the current algorithm and modified algorithm was 66.08% and 64.94%, respectively. There is no significant difference in overall match rates between the two algorithms at the 0.05 level.

## Conclusions

Medical residency positions are competitive and challenging. Research is gradually providing insights on how to improve the recruitment, management, and well-being of residents and their patients. Candidates have been found to favor an 8-9am start time, four-hour duration, utilizing one-on-one interviews, lasting 15–30 minutes each [34]. Though virtual interviews may mitigate their high cost of travel and time, the majority of medical students and residents still prefer in-person interviews but feel that virtual interviews should be retained as an option in some circumstances [35]. Utilization of a team night shift system instead of the traditional one in four nights call may improve resident well-being and daytime teaching and feedback [36]. Covid-19 has had a direct personal impact on resident’s specialty choice due to concerns about frontline work, work-life balance, and risk of harm [37]. Though telemedicine has been increasing due to Covid-19, residents revealed feeling less confident in managing chronic diseases through telemedicine visits, and prefer in-person visits during their training, and would prefer less than 50% of visits to be telemedicine in their future careers [38]. Elimination of the extended duration work shifts of greater than 24 hours commonly worked by interns have been found to reduce the rate of significant medical errors, attention failures, and adverse medical events [39]. Building on this research stream, this study examined the algorithm used to recruit medical graduates to residency positions, where concerns have been raised about its effectiveness, transparency, and fairness.

This study in the medical residency match context showed how two challenges of bias verification and algorithm modification to reduce the bias may be overcome despite little information available. The study first used randomized computer-generated data for the inputs–student and program rankings–that were consistent with their publicly disclosed aggregate mean and range statistics. It then compiled the algorithm’s steps using publicly reported descriptions of the algorithm. It ran thousands of simulations of the current algorithm using the computer-generated data to obtain match outcomes. These match outcomes were then logistically regressed against the data inputs of two stakeholders–graduate medical applicants and programs–to examine their explanatory power. The use of multiple logistical regression including linear and non-linear control variables provides a more stringent for the existence or absence of a relationship with the dependent variable than a bivariate analysis.

### Discussion of the findings

The study found that the current algorithm, which has been in use for over a generation, is related to programs’ input but not applicants’ inputs. The students’ rankings of positions was found unrelated to match outcomes, contrary to the intended purpose of current algorithm that seeks to prioritize the preferences of applicants, and place them in the most preferred program possible on their rank ordered list. The study provides additional evidence from the medical residency context, which has used the same algorithm for over a generation, that the existence of biased algorithms may be widespread.

After having observed bias in the current algorithm, we modified the algorithm’s order of procedures to make student rankings have a primary influence. When inputing the same data, the modified algorithm resulted in significant influences by both stakeholders–applicants and programs–suggesting that the matching algorithm can be improved and result in a positive sum, rather than a zero-sum, win-lose situation among stakeholders. The overall match performance, in terms of the percentage of applicants matched to programs, was not significantly different between the current and modified algorithms.

### Framework for stakeholders responsiveness

The findings of the study helped develop a framework shown in Table 4 for evaluating an algorithm’s responsiveness to various stakeholders. The table summarizes the detailed inner workings of an algorithm that often defy comprehension. The left column shows an algorithm’s three phases–initial, middle, and final–shown in the left column. The middle and right columns show the participation of various stakeholders in the two algorithms.

**Table 4:** Increasing algorithm’s responsiveness to multiple stakeholders (programs and students).

Dynamic Stages	Algorithm	
	Existing	Modified
**Initial—Focus**	Student’s ranking	Joint (Student’s ranking, Program’s ranking)
**Middle–Primary criterion**	Program’s ranking	Student’s ranking
**Final—Tie Breaking criterion**	Program ranking	Program’s ranking
**Number of steps**	More—because initial matches are tentative	Fewer—because matches are final

The initial phase shows what an algorithm considers first and displays the face of the algorithm. This initial face may be deceiving because the outcomes are mostly determined by procedures in the middle and final phases. The current matching algorithm is described as student-proposed, but matches so derived are tentative, and students may be dematched from a program by later procedures. In contrast, the modified algorithm incorporates from the start the joint ratings of both students and programs.

The middle phase of the algorithm shows the primary screen used for processing the majority of the cases. The current algorithm’ main screen is the program’s ranking of students, while the modified algorithm employs the student’s ranking of the program.

The final phase shows how the last residency spot in a program is decided when a tie among students exists. In both algorithms, the ties are broken by the program’s ranking of students.

For the algorithm to be more inclusive, participation of multiple stakeholders is desirable in a phase and across phases. The current algorithm has only students participating in the initial phase, and only programs are active in the middle and final phases. In the modified algorithm, both students and program rankings are taken into account in the initial phase, students are active in the middle phase, and programs are active in the final phase, suggesting joint and interweaving participation over time.

Lastly, the transparency of the algorithm is influenced by its number of steps. Complex algorithms go through more steps and reduce public understanding. In the current algorithm, initial matches proposed by a student are tentative and can be reversed by later procedures, increasing the number of steps it goes through for each applicant. In contrast, the modified algorithm considers jointly all students and programs, and arrives at final matches with fewer steps in a simpler, more transparent way.

### Main contributions

A validation examination of the existing residency matching algorithm suggests that it does not achieve what it purports to do–prioritize the preferences of applicants, and place them in the most preferred program possible on their rank ordered list. Its initial tentative matches are proposed by student applicants, but student ranking are not related to the final match outcomes. The match outcomes are related to input program rankings. The current algorithm goes through many iterative steps as initial matches may be dematched reducing its transparency. The modified algorithm, in which student preferences are primary, results in matches that are related to the inputs of both students and programs, arrives at final matches with fewer iterative steps, and is more transparent and inclusive.

### Literature feedback

The literature has noted that along with the growth and prevalence of algorithms, concerns have arisen about whether they also create, perpetuate, and exacerbate social inequities among affected groups. Biases may arise in an algorithm’s data inputs, processes, or application. Some stakeholders that are affected by the algorithm often have limited access to the data which may be proprietary and confidential, as well as have limited access to the code and little understanding of how an algorithm’s procedures work. Consequently, stakeholders face formidable challenges in validating the algorithm, identifying, and proving that a bias exists, and then modifying the algorithm in a way that reduces the bias.

This study’s findings provide evidence that bias can exist even in a longstanding and widely used algorithm, that an algorithm may not deliver on its intended purpose, and that it can favor one stakeholder and disadvantage another. While data inaccessibility and opaque algorithm procedures pose difficulties, this study showed novel ways of overcoming them through randomizing data inputs and using the limited descriptive information on an algorithm’s procedures to replicate the algorithm and conduct an external verification. The study also showed that by modifying the order of its procedures, an algorithm can be improved to satisfy more stakeholders.

### Policy recommendations

Algorithms are difficult to access, understand, monitor, and change. Therefore, policymakers, such as government regulators or industry professional associations, should take an active role and require multiple stakeholders be engaged early in the design of the algorithm, External independent evaluators, with technical expertise, must be given access to de-identified data inputs and to the algorithms procedures, and must periodically monitor that the algorithm delivers on its intended purpose and that its benefits are equitably distributed across stakeholders. When benefits are not equitably distributed, the algorithm’s procedures should be modified to improve the distribution of benefits across stakeholders.

### Limitations and future work

Additional research is needed on how to design algorithms from the outset that meet multiple stakeholders’ interests. How to fine-tune an algorithm’s outputs to optimize or equalize inclusion is another area needing research. This study examined one algorithm at a time. Research is needed on how to combine inputs of multiple stakeholders and on the combination of the outputs of multiple algorithms. This study did not examine how to implement and integrate a modified algorithm into an organization. Future research is needed on how to implement in organizations algorithm changes that improve inclusion of multiple stakeholders.
